# Special function form solutions of multi-parameter generalized Mittag-Leffler kernel based bio-heat fractional order model subject to thermal memory shocks

**DOI:** 10.1371/journal.pone.0299106

**Published:** 2024-03-08

**Authors:** Muhammad Bilal Riaz, Aziz Ur Rehman, Jan Martinovic, Muhammad Abbas

**Affiliations:** 1 IT4Innovations, VSB—Technical University of Ostrava, Ostrava, Czech Republic; 2 Department of Computer Science and Mathematics, Lebanese American University, Byblos, Lebanon; 3 Department of Mathematics, University of Management and Technology, Lahore, Pakistan; 4 Department of Mathematics, University of Sargodga, Sargodga, Pakistan; University of Education, PAKISTAN

## Abstract

The primary objective of this research is to develop a mathematical model, analyze the dynamic occurrence of thermal shock and exploration of how thermal memory with moving line impact of heat transfer within biological tissues. An extended version of the Pennes equation as its foundational framework, a new fractional modelling approach called the Prabhakar fractional operator to investigate and a novel time-fractional interpretation of Fourier’s law that incorporates its historical behaviour. This fractional operator has multi parameter generalized Mittag-Leffler kernel. The fractional formulation of heat flow, achieved through a generalized fractional operator with a non-singular type kernel, enables the representation of the finite propagation speed of heat waves. Furthermore, the dynamics of thermal source continually generates a linear thermal shock at predefined locations within the tissue. Introduced the appropriate set of variables to transform the governing equations into dimensionless form. Laplace transform (LT) is operated on the fractional system of equations and results are presented in series form and also expressed the solution in the form of special functions. The article derives analytical solutions for the heat transfer phenomena of both the generalized model, in the Laplace domain, and the ordinary model in the real domain, employing Laplace inverse transformation. The pertinent parameter’s influence, such as *α*, *β*, *γ*, *a*_0_, *b*_0_, to gain insights into the impact of the thermal memory parameter on heat transfer, is brought under consideration to reveal the interesting results with graphical representations of the findings.

## Introduction

In this contemporary era of innovations and development of biological sciences, bio-heat transfer stands as a captivating and multidisciplinary exploration that have to be a great importance for researchers and scientists for last few decades because it effectively used in various fields of the dynamics of heat propagation within living organisms. This intriguing field of study encompasses a diverse range of phenomena, such as the conveyance of heat within cells and tissues, as well as the mechanisms responsible for thermoregulation, enabling animals to regulate their body temperatures. Bioheat transfer delves into the fundamental principles of fluid mechanics, thermodynamics, and physiological processes to gain a comprehensive understanding of the intricate interplay between heat and life. In this context, bio-heat transfer flow is one of the most prominent fields, and in recent times, there have been significant advancements in this domain. In the field of Bioheat transfer, it is analysed that how heat moves and distributed in the biological systems. For smooth functionality of living tissues heat plays a pivotal role, which is a form of energy. The mathematical model of heat transfer proposed by Pennes [1], in 1948, has been widely studied in different circumstances. The Pennes bioheat equation used to model heat transfer in live biological bodies to analyze cell’s metabolism and blood perfusion effects. Heat exerts profound effects on numerous physiological processes, including the regulation of enzymatic reactions and the control of metabolic activities. To gain a comprehensive grasp of how these systems function, it is imperative to possess a thorough comprehension of how heat is generated, conveyed, and dissipated within biological contexts. In this dynamic and rapidly evolving field, researchers employ a encompassing theoretical models, multifaceted approach, experimental methodologies, and computer simulations, to unravel the intricate intricacies of heat transfer within biological systems. This interdisciplinary collaboration bridges the gaps between biologists, engineers, physicists, mathematicians, and medical professionals [2–5], fostering a richer understanding of the subject matter. Being mindful of its important properties, features and abilities, the wide application of this model is observed in biological science such as plasma, handling of biological fluid, blood, etc. According to the prevailing modern scientific challenges, several mathematicians, researchers, scientists and engineers pay extraordinary attention to biology, engineering, chemistry, specially focus to study the Pennes bioheat equation due to its importance in biological contexts. Ragab et al. [6] provides an analytical solution to the modified Pennes bioheat conduction equation with a single relaxation time, and also investigate the thermal reactions caused by temperature shock, specifically the influence of heat generation through heat treatment on a skin tumor.

The sudden changes, rapid and substantial fluctuations in temperature is a reason of producing thermal shock in a biological systems. In the realm of bioheat transfer, thermal shock represents the adverse consequences brought about by substantial fluctuations in temperature transformation in biological family. Grasping the underlying physics and ramifications of thermal shock holds paramount significance across various domains, including biomedical engineering, cryobiology, thermal medicine, and thermal therapy [7, 8]. Different techniques have been applied to propose an analytical solutions of bioheat equations. Zhou et al. [9] estimated the temperature distribution in one dimensional Cartesian coordinates to analyzed the biological tissues at steady-state. The same analysis was presented in Minhua et al. [10] by considering the temperature distribution in cylindrical geometry. Shih et al. [11] depicted and explained the results obtained from the living tissues models with employing sinusoidal heat flux on skin surface. Fluid flow with thermal shock involves the sudden exposure of a fluid system to extreme temperature variations, inducing rapid thermal expansions or contractions are investigated by Fuzhang et al. [12]. For the estimation of the localized and concentrated heat transfer in the spherical system regarding time dependent and analytical analysis of bioheat equation was presented and discussed by Gutierrez [13] that required for the ablation of the cancerous cells. According to Animasaun et al. [14], thermal shock refers to the rapid and extreme temperature changes experienced by a material, causing it to undergo stress and potentially crack or break. This phenomenon occurs when a substance undergoes abrupt temperature variations, leading to differential expansion or contraction within the material. Similar studies on Pennes bioheat transfer equation recorded in literature [15, 16] and references therein.

The versatile and valuable impacts of fractional calculus in the field of electrical engineering, electrochemistry, control theory, electromagnetism, mechanics, image processing, bioengineering, physics, finance, fluid dynamics, and many others make it a valuable tool for study. Fractional derivatives not only keep the record of the present but also the past, so they are very suitable and accurate when the system has long-term memory. It has several applications in physical science as well as in other areas such as biology, astrophysics, ecology, geology and chemistry. The mechanism of non-Newtonian models is elaborated successfully with the fractional calculus in the past decades due to its simple and elegant description of the complexity of its behavior. One of the important feature and most commonly known name of non-Newtonian fluid is viscoelastic fluid that which exhibit the behaviour of elasticity and viscosity. Such types of fluid models have great implications in various fields namely polymerization, industrial as well as mechanical engineering and also in the field of auto mobile industry due to its significance. Fractional calculus is very helpful in the interpretation of the viscoelastic nature of the materials. Taking into account the enormous mentioned properties, many researchers paid attention to analyse the fractional behaviour of different fluid models directly or indirectly in case of derivatives when it is considered as non-integer order from. Instantaneous heat conduction and local thermal equilibrium are presupposed in these models [17–20]. Bagley and Torvik [21] noted the fractional calculus application on the viscoelastic fluids. Rehman et al. [22] examined the fractional Maxwell fluid and explored the closed solution of shear stress and velocity. Anouar et al. [23] presented a new time-fractional heat conduction model with three-phase-lags and three distinct fractional-order derivatives. Riaz et al. [24] analyzed the influence of the MHD on the heattransfer of fractionalized Oldroyd-B fluid. Some features of Maxwell fluid along with observing the impact of Newtonian Heating and developed the fractional model using Prabhakar fractional approach is explored by Rehman et al. [25]. Abouelregal et al. [26] developed a fractional heat conduction models with phase lags and Euler–Bernoulli beam theory are used to derive the governing system equations by employing Atangana-Baleanu fractional operator. The ABC, CF and CPC comparative analysis of second-grade fluid under Newtonian heating effect, found the series solution, performed by Rehman et al. [27]. Some of the contributions related to fractional integral operators in bioheat transfer models are highlighted in [28–34].

In this paper, the derivation of the general analytic solution for the fractional version of original mathematical model of the temperature gradient through the application of famous derivative operator such as Prabhaker operator is conducted by adopting the Pennes’ bioheat transfer equation, and calculated the result by jointly applied the method of Laplace transformation. It is important to highlight, that the presented mathematical model is novel in the field’s existing body of literature, because this model pertains to the nature of the heat source. The formulation presented which permits the application of a thermal shock in specific regions of these tissues at some particular time points. This characteristic possesses the potential for diverse applications in various medical treatment contexts. But it is noticed that the Pennes’ bioheat temperature equation with this innovative fractional operator namely Prabhakar fractional operator having generalized multi-parameter Mittag-Leffler kernel are not investigated, and not available in the previous literature related to fluid mechanics of fractional models. Inspired by the above literature, this article is devoted to studying the heat transfer analysis of the Pennes’ bioheat fractionalized temperature distribution with initial/boundary conditions, and to obtain the analytical solution of Pennes’ bioheat equation. We have converted the integer-order derivative Pennes’ bioheat model with the non-integer order derivative Prabhakar model. Laplace transform have been employed to get the analytical solutions of the current problem. The non-dimensional temperature phenomenon is solved by the integral LT. However, the expression for the non-dimensional temperature in the Laplace domain obtained in order to find the analytical solution via Prabhakar approach are more complex, due to the complexity of such expression, it is a big challenge to finding the inverse Laplace transform. Therefore, the obtained results are presented in a series form and also in the form of generalized special functions namely Mittag-Leffler function. Such exact solutions have never been noted in the literature before and the derived analytical expression are unique. Hence this article makes valuable contributions to the existing literature in view of pan-city of exact solutions of Pennes’ bioheat model with suitable conditions. The influence of embedded parameters, such as *α*, *a*_0_, *β*, *b*_0_, *γ*, to gain insights into the impact of the thermal memory parameter on heat transfer, is brought under consideration to reveal the interesting results with the assistance of graphs.

## Mathematical model

**Fig 1:**
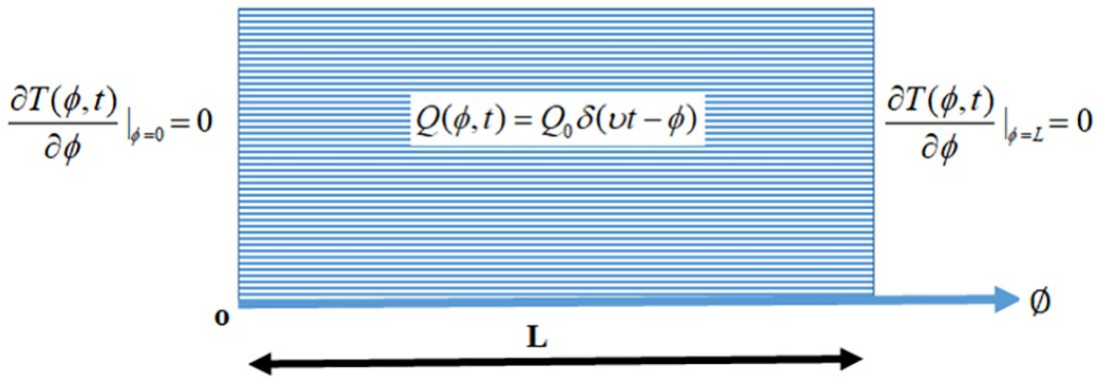
Schematic drawing of the flow model.

The phenomena of heat transfer in one-dimensional biological tissue with a length of L in the direction of *φ*-axis, is examined here, which is exposed to moving thermal shocks (as shown in Fig 1). The relative temperature *T*(*φ*, *t*) is governed with initial and boundary conditions [1, 35, 36] of Pennes’ bioheat equation will take the form:

$$\begin{array}{c} \rho_{t}C_{t}\frac{\partial T(\varphi ,t)}{\partial t}=-\frac{\partial q(\varphi ,t)}{\partial \varphi }+\rho_{b}C_{b}w_{b}\left[T_{a}-T\left(\varphi ,t\right)\right]+Q_{0}\delta \left(\upsilon t-\varphi \right)+Q_{m}, \end{array}$$ (1)

$$\begin{array}{c} q\left(\varphi ,t\right)=-k_{t}\frac{\partial T(\varphi ,t)}{\partial \varphi },\;\left(Fourier^{\prime }s\;Law\right), \end{array}$$ (2)

where *δ*(.) represents Dirac function, *Q*_0_*δ*(*υt* − *φ*) denotes the special heat source term at any time t while in the direction of *φ* = *υt* a constant heat source *Q*_0_ is applied, where in the above equations *υ* > 0 parameter is used that represent the velocity of moving heat transfer. Also, the following conditions are considered for Eqs 1 and 2:

$$\begin{array}{c} T\left(\varphi ,0\right)=T_{a},\;\frac{\partial T(\varphi ,t)}{\partial \varphi }{{|}_{\varphi =0}=0,\;and\;\frac{\partial T(\varphi ,t)}{\partial \varphi }|}_{\varphi =L}=0. \end{array}$$ (3)

Introducing the new quantities used here to non-dimensionalize the equations:

$$\begin{array}{cc} & t^{*}=\frac{\eta_{t}t}{L^{2}},\;\varphi^{*}=\frac{\varphi }{L},\;\eta_{t}=\frac{k_{t}}{C_{t}\rho_{t}},\;T^{*}=\frac{T-T_{a}}{T_{a}},\;Q_{0}=\frac{Q_{0}}{\upsilon T_{a}C_{t}\rho_{t}},\\ & q^{*}=\frac{Lq}{T_{a}k_{t}},\;Q_{m}=\frac{L^{2}Q_{m}}{T_{a}k_{t}}\chi^{*}=\frac{\chi }{\chi_{0}},\;a_{0}=\frac{w_{b}C_{b}\rho_{b}L^{2}}{\eta_{t}C_{t}\rho_{t}},\;b_{0}=\frac{\eta_{t}}{L\upsilon }. \end{array}$$ (4)

When using the newly introduced entities as defined in the above relation Eq 4 and replacing these entities into Eqs 1 and 2, and after that * symbol ignoring, then finally the equations transformed in the following form:

$$\begin{array}{c} \frac{\partial T(\varphi ,t)}{\partial t}=-\frac{\partial q(\varphi ,t)}{\partial \varphi }-a_{0}T\left(\varphi ,t\right)+Q_{0}\delta \left(t-b_{0}\varphi \right)+Q_{m}, \end{array}$$ (5)

$$\begin{array}{c} q\left(\varphi ,t\right)=-\frac{\partial T(\varphi ,t)}{\partial \varphi }, \end{array}$$ (6)

Along with the set of non-dimensional from of conditions becomes

$$\begin{array}{cc} & T(\varphi ,0)=0,\;0\leq \varphi \leq 1\\ & \frac{\partial T(\varphi ,t)}{\partial \varphi }{{|}_{\varphi =0}=0,\;and\;\frac{\partial T(\varphi ,t)}{\partial \varphi }|}_{\varphi =1}=0, \end{array}$$ (7)

## Preliminaries

The Prabhakar time derivative fractional operator employed in the considered work, that is developed by Prabhakar et al. [37] and mathematical description of this novel regularised Prabhakar derivativ is expressed as:

Dα,ζ,ηγht=Eα,m−ζ,η−γhmt=∫0tt−σm−ζ−1Eα,m−ζ−γηt−σαhmσdσ.c (8)

and

$$\begin{array}{c} E_{\alpha ,\zeta ,\eta }^{\gamma }h\left(t\right)=\int_{0}^{t}{\left(t-\sigma \right)}^{\zeta -1}E_{\alpha ,\zeta }^{\gamma }\left(\eta {\left(t-\sigma \right)}^{\alpha }\right)h\left(\sigma \right)d\sigma . \end{array}$$

is known as Prabhakar integrall and

$$\begin{array}{c} E_{\alpha ,\zeta }^{\gamma }\left(℘\right)=\sum_{n=0}^{\infty }\frac{\Gamma \left(\gamma +n\right){℘}^{n}}{n!\Gamma (\gamma )\Gamma (\alpha n+\zeta )},\;℘,\alpha ,\zeta ,\gamma \in C,\;Re\left(\alpha \right)>0 \end{array}$$

represents the multi-parameter Mittag-Leffler function. Also, it is noted that the function $t^{\zeta -1}E_{\alpha ,\zeta }^{\gamma }\left(\eta t^{\alpha }\right)$ with *Re*(*α*) > 0, t∈R and α,ζ,γ,η∈C, is known as kernel of this novel operator.

This generalized operator has Laplace transform in the following form:

$$\begin{array}{c} L{\{}^{C}D_{\alpha ,\zeta ,\eta }^{\gamma }h(t)\}=S^{\zeta -m}{\left(1-\eta S^{-\alpha }\right)}^{\gamma }L\{h^{\left(m\right)}(t)\}. \end{array}$$ (9)

Here Laplace transform parameter is denoted by *S* and fractional parameters are denoted by *α*, *ζ*, *γ*.

## Fractional formulation of the model

The generalised Fourier laws based on Prabhakar’s derivative operator are written as:

$$\begin{array}{c} q\left(\varphi ,t\right)={-}^{C}D_{\alpha ,\beta ,℘}^{\gamma }\frac{\partial T(\varphi ,t)}{\partial \varphi }, \end{array}$$ (10)

Where DCα,β,℘γ.,. denotes the Prabhakar derivative operator, for further properties about the novel fractional operator are discussed in [37]. Also, when taking *β* = *γ* = 0, we obtained the classical Fourier’s law.

### Investigation of exact solution for stated generalised bio-heat equation

Implement the definition of Laplace operator on Eqs 5 and 6 with transformed conditions as mentioned in Eq 7, we will get

$$\begin{array}{c} S\bar{T}\left(\varphi ,S\right)=-\frac{\partial \bar{q}\left(\varphi ,S\right)}{\partial \varphi }-a_{0}\bar{T}\left(\varphi ,S\right)+Q_{0}e^{-b_{0}\varphi S}+\frac{1}{S}Q_{m}, \end{array}$$ (11)

and

$$\begin{array}{c} \bar{q}\left(\varphi ,S\right)=-S^{\beta }{\left(1-℘S^{-\alpha }\right)}^{\gamma }\frac{\partial \bar{T}\left(\varphi ,S\right)}{\partial \varphi }. \end{array}$$ (12)

with transformed boundary conditions

$$\begin{array}{c} \bar{T}\left(\varphi ,0\right)=0,\;\frac{\partial \bar{T}\left(\varphi ,S\right)}{\partial \varphi }{{|}_{\varphi =0}=0,\;and\;\frac{\partial \bar{T}\left(\varphi ,S\right)}{\partial \varphi }|}_{\varphi =1}=0. \end{array}$$ (13)

Employing the definition of Laplace operator, by substituting Eq 12 into Eq 11, then the energy equation is obtained as:

$$\begin{array}{cc} \frac{S}{S^{\beta }{\left(1-℘S^{-\alpha }\right)}^{\gamma }}\bar{T}\left(\varphi ,S\right) & =\frac{\partial^{2}\bar{T}\left(\varphi ,S\right)}{\partial \varphi^{2}}-\frac{a_{0}}{S^{\beta }{\left(1-℘S^{-\alpha }\right)}^{\gamma }}\bar{T}\left(\varphi ,S\right)\\ & +\frac{Q_{0}}{S^{\beta }{\left(1-℘S^{-\alpha }\right)}^{\gamma }}e^{-b_{0}\varphi S}+\frac{1}{SS^{\beta }{\left(1-℘S^{-\alpha }\right)}^{\gamma }}Q_{m}, \end{array}$$ (14)

$$\begin{array}{c} \frac{\partial^{2}\bar{T}\left(\varphi ,S\right)}{\partial \varphi^{2}}=\frac{S+a_{0}}{S^{\beta }{\left(1-℘S^{-\alpha }\right)}^{\gamma }}\bar{T}\left(\varphi ,S\right)-\frac{1}{S^{\beta }{\left(1-℘S^{-\alpha }\right)}^{\gamma }}[Q_{0}e^{-b_{0}\varphi S}+\frac{Q_{m}}{S}], \end{array}$$ (15)

The temperature solution of Eq 15 is calculated as:

$$\begin{array}{ccc} \bar{T}\left(\varphi ,S\right) & & =e_{1}e^{-\varphi \sqrt{\frac{S+a_{0}}{S^{\beta }{\left(1-℘S^{-\alpha }\right)}^{\gamma }}}}+e_{2}e^{\varphi \sqrt{\frac{S+a_{0}}{S^{\beta }{\left(1-℘S^{-\alpha }\right)}^{\gamma }}}}\\ & & +\frac{Q_{0}e^{-b_{0}\varphi S}}{\left(S+a_{0}\right)-b_{0}^{2}S^{\beta +2}{\left(1-℘S^{-\alpha }\right)}^{\gamma }}+\frac{Q_{m}}{S(S+a_{0})}. \end{array}$$ (16)

Applying the transformed boundary conditions, then energy equation has solution as:

$$\begin{array}{cc} & \bar{T}\left(\varphi ,S\right)\\ & =[\frac{b_{0}SQ_{0}e^{-\varphi \sqrt{\frac{S+a_{0}}{S^{\beta }{\left(1-℘S^{-\alpha }\right)}^{\gamma }}}}}{(\left(S+a_{0}\right)-b_{0}^{2}S^{\beta +2}{\left(1-℘S^{-\alpha }\right)}^{\gamma })\sqrt{\frac{S+a_{0}}{S^{\beta }{\left(1-℘S^{-\alpha }\right)}^{\gamma }}}}][\frac{e^{-b_{0}S}-e^{\varphi \sqrt{\frac{S+a_{0}}{S^{\beta }{\left(1-℘S^{-\alpha }\right)}^{\gamma }}}}}{e^{\varphi \sqrt{\frac{S+a_{0}}{S^{\beta }{\left(1-℘S^{-\alpha }\right)}^{\gamma }}}}-e^{-\varphi \sqrt{\frac{S+a_{0}}{S^{\beta }{\left(1-℘S^{-\alpha }\right)}^{\gamma }}}}}]\\ & +[\frac{b_{0}SQ_{0}e^{\varphi \sqrt{\frac{S+a_{0}}{S^{\beta }{\left(1-℘S^{-\alpha }\right)}^{\gamma }}}}}{(\left(S+a_{0}\right)-b_{0}^{2}S^{\beta +2}{\left(1-℘S^{-\alpha }\right)}^{\gamma })\sqrt{\frac{S+a_{0}}{S^{\beta }{\left(1-℘S^{-\alpha }\right)}^{\gamma }}}}][\frac{e^{-b_{0}S}-e^{-\varphi \sqrt{\frac{S+a_{0}}{S^{\beta }{\left(1-℘S^{-\alpha }\right)}^{\gamma }}}}}{e^{-\varphi \sqrt{\frac{S+a_{0}}{S^{\beta }{\left(1-℘S^{-\alpha }\right)}^{\gamma }}}}-e^{\varphi \sqrt{\frac{S+a_{0}}{S^{\beta }{\left(1-℘S^{-\alpha }\right)}^{\gamma }}}}}]\\ & +[\frac{Q_{0}e^{-b_{0}\varphi S}}{(\left(S+a_{0}\right)-b_{0}^{2}S^{\beta +2}{\left(1-℘S^{-\alpha }\right)}^{\gamma })\sqrt{\frac{S+a_{0}}{S^{\beta }{\left(1-℘S^{-\alpha }\right)}^{\gamma }}}}]+\frac{Q_{m}}{S(S+a_{0})}. \end{array}$$ (17)

This complicated expression can be written as:

$$\begin{array}{cc} \bar{T}\left(\varphi ,S\right) & =\bar{E}\left(S\right)[\frac{e^{-b_{0}S}-e^{\varphi \sqrt{A(S)}}}{e^{\varphi \sqrt{A(S)}}-e^{-\varphi \sqrt{A(S)}}}]e^{-\varphi \sqrt{A(S)}}+\bar{E}\left(S\right)[\frac{e^{-b_{0}S}-e^{-\varphi \sqrt{A(S)}}}{e^{-\varphi \sqrt{A(S)}}-e^{\varphi \sqrt{A(S)}}}]e^{\varphi \sqrt{A(S)}}\\ & +\bar{B}\left(S\right)e^{-b_{0}\varphi S}+\bar{D}\left(S\right). \end{array}$$ (18)

To transform the solution in time variable again, we have to employ inverse Laplace transformation technique on Eq 18, but it is very complicated and to find its analytical solution, we have to find Laplace inverse of each term, then using convolution property to obtain the solution in final form. Now, simplified the expression first as:

$$\begin{array}{cc} E(t) & =L^{-1}\{\bar{E}\left(S\right)\},\\ & =L^{-1}\{\frac{b_{0}SQ_{0}}{(\left(S+a_{0}\right)-b_{0}^{2}S^{\beta +2}{\left(1-℘S^{-\alpha }\right)}^{\gamma })\sqrt{\frac{S+a_{0}}{S^{\beta }{\left(1-℘S^{-\alpha }\right)}^{\gamma }}}}\},\\ & =L^{-1}\{\sum_{m=0}^{\infty }b_{0}^{1+2m}Q_{0}\frac{S^{1+\beta /2+\beta m+2m}}{{\left(1-℘S^{-\alpha }\right)}^{\gamma (m+1/2)}}\frac{1}{{\left(S+a_{0}\right)}^{\left(m+3/2\right)}}\},\\ & =\sum_{m=0}^{\infty }b_{0}^{1+2m}Q_{0}t^{-(m+1/2)(\beta +2)-1}E_{\alpha ,-(m+1/2)(\beta +2)}^{-\gamma (m+1/2)}\left(℘t^{\alpha }\right)*e^{-a_{0}t}t^{m+1/2}, \end{array}$$ (19)

$$\begin{array}{cc} N(t) & =L^{-1}\{\bar{N}\left(S\right)\},\\ & =L^{-1}\{[\frac{e^{-b_{0}S}-e^{\varphi \sqrt{\frac{S+a_{0}}{S^{\beta }{\left(1-℘S^{-\alpha }\right)}^{\gamma }}}}}{e^{\varphi \sqrt{\frac{S+a_{0}}{S^{\beta }{\left(1-℘S^{-\alpha }\right)}^{\gamma }}}}-e^{-\varphi \sqrt{\frac{S+a_{0}}{S^{\beta }{\left(1-℘S^{-\alpha }\right)}^{\gamma }}}}}]e^{-\varphi \sqrt{\frac{S+a_{0}}{S^{\beta }{\left(1-℘S^{-\alpha }\right)}^{\gamma }}}}\},\\ & =L^{-1}\{\sum_{k=0}^{\infty }e^{-b_{0}S}e^{-\left(1+2k+\varphi \right)\sqrt{\frac{S+a_{0}}{S^{\beta }{\left(1-℘S^{-\alpha }\right)}^{\gamma }}}}-\sum_{k=0}^{\infty }e^{-\left(2k+\varphi \right)\sqrt{\frac{S+a_{0}}{S^{\beta }{\left(1-℘S^{-\alpha }\right)}^{\gamma }}}}\},\\ & =L^{-1}\{e^{-b_{0}S}\bar{N_{1}}\left(S\right)-\bar{N_{2}}\left(S\right)\},\\ & =N_{1}\left(t-b_{0}\right)u\left(t-b_{0}\right)-N_{2}\left(t\right), \end{array}$$ (20)

where

$$\begin{array}{cc} N_{1}\left(t\right) & =L^{-1}\{\bar{N_{1}}\left(S\right)\},\\ & =L^{-1}\{\sum_{k=0}^{\infty }e^{-\left(1+2k+\varphi \right)\sqrt{\frac{S+a_{0}}{S^{\beta }{\left(1-℘S^{-\alpha }\right)}^{\gamma }}}}\},\\ & =L^{-1}\{\sum_{k=0}^{\infty }\sum_{n=0}^{\infty }\sum_{m=0}^{n}\frac{{\left(-1\right)}^{n}{\left(1+2k+\varphi \right)}^{n}{\left(a_{0}\right)}^{n/2-m}\Gamma \left(n/2+1\right)}{n!m!\Gamma (n/2-m+1)}\frac{1}{S^{\left(\beta n/2-m\right)}{\left(1-℘S^{-\alpha }\right)}^{\gamma n/2}}\},\\ & =\sum_{k=0}^{\infty }\sum_{n=0}^{\infty }\sum_{m=0}^{n}\frac{{\left(-1\right)}^{n}{\left(1+2k+\varphi \right)}^{n}{\left(a_{0}\right)}^{n/2-m}\Gamma \left(n/2+1\right)}{n!m!\Gamma (n/2-m+1)}t^{\beta n/2-m-1}E_{\alpha ,\beta n/2-m}^{\gamma n/2}\left(℘t^{\alpha }\right),\\ N_{2}\left(t\right) & =L^{-1}\{\bar{N_{2}}\left(S\right)\},\\ & =L^{-1}\{\sum_{k=0}^{\infty }e^{-\left(2k+\varphi \right)\sqrt{\frac{S+a_{0}}{S^{\beta }{\left(1-℘S^{-\alpha }\right)}^{\gamma }}}}\},\\ & =L^{-1}\{\sum_{k=0}^{\infty }\sum_{n=0}^{\infty }\sum_{m=0}^{n}\frac{{\left(-1\right)}^{n}{\left(2k+\varphi \right)}^{n}{\left(a_{0}\right)}^{n/2-m}\Gamma \left(n/2+1\right)}{n!m!\Gamma (n/2-m+1)}\frac{1}{S^{\left(\beta n/2-m\right)}{\left(1-℘S^{-\alpha }\right)}^{\gamma n/2}}\},\\ & =\sum_{k=0}^{\infty }\sum_{n=0}^{\infty }\sum_{m=0}^{n}\frac{{\left(-1\right)}^{n}{\left(2k+\varphi \right)}^{n}{\left(a_{0}\right)}^{n/2-m}\Gamma \left(n/2+1\right)}{n!m!\Gamma (n/2-m+1)}t^{\beta n/2-m-1}E_{\alpha ,\beta n/2-m}^{\gamma n/2}\left(℘t^{\alpha }\right), \end{array}$$ (21)

$$\begin{array}{cc} R(t) & =L^{-1}\{\bar{R}\left(S\right)\},\\ & =L^{-1}\{[\frac{e^{-b_{0}S}-e^{-\varphi \sqrt{\frac{S+a_{0}}{S^{\beta }{\left(1-℘S^{-\alpha }\right)}^{\gamma }}}}}{e^{\varphi \sqrt{\frac{S+a_{0}}{S^{\beta }{\left(1-℘S^{-\alpha }\right)}^{\gamma }}}}-e^{-\varphi \sqrt{\frac{S+a_{0}}{S^{\beta }{\left(1-℘S^{-\alpha }\right)}^{\gamma }}}}}]e^{\varphi \sqrt{\frac{S+a_{0}}{S^{\beta }{\left(1-℘S^{-\alpha }\right)}^{\gamma }}}}\},\\ & =L^{-1}\{\sum_{k=0}^{\infty }e^{-b_{0}S}e^{\left(\varphi -2k-1\right)\sqrt{\frac{S+a_{0}}{S^{\beta }{\left(1-℘S^{-\alpha }\right)}^{\gamma }}}}-\sum_{k=0}^{\infty }e^{\left(\varphi -2k-2\right)\sqrt{\frac{S+a_{0}}{S^{\beta }{\left(1-℘S^{-\alpha }\right)}^{\gamma }}}}\},\\ & =L^{-1}\{e^{-b_{0}S}\bar{R_{1}}\left(S\right)-\bar{R_{2}}\left(S\right)\},\\ & =R_{1}\left(t-b_{0}\right)u\left(t-b_{0}\right)-R_{2}\left(t\right), \end{array}$$ (22)

where

$$\begin{array}{cc} R_{1}\left(t\right) & =L^{-1}\{\bar{R_{1}}\left(S\right)\},\\ & =L^{-1}\{\sum_{k=0}^{\infty }e^{\left(\varphi -2k-1\right)\sqrt{\frac{S+a_{0}}{S^{\beta }{\left(1-℘S^{-\alpha }\right)}^{\gamma }}}}\},\\ & =L^{-1}\{\sum_{k=0}^{\infty }\sum_{n=0}^{\infty }\sum_{m=0}^{n}\frac{{\left(\varphi -2k-1\right)}^{n}{\left(a_{0}\right)}^{n/2-m}\Gamma \left(n/2+1\right)}{n!m!\Gamma (n/2-m+1)}\frac{1}{S^{\left(\beta n/2-m\right)}{\left(1-℘S^{-\alpha }\right)}^{\gamma n/2}}\},\\ & =\sum_{k=0}^{\infty }\sum_{n=0}^{\infty }\sum_{m=0}^{n}\frac{{\left(\varphi -2k-1\right)}^{n}{\left(a_{0}\right)}^{n/2-m}\Gamma \left(n/2+1\right)}{n!m!\Gamma (n/2-m+1)}t^{\beta n/2-m-1}E_{\alpha ,\beta n/2-m}^{\gamma n/2}\left(℘t^{\alpha }\right),\\ R_{2}\left(t\right) & =L^{-1}\{\bar{R_{2}}\left(S\right)\},\\ & =L^{-1}\{\sum_{k=0}^{\infty }e^{\left(\varphi -2k-2\right)\sqrt{\frac{S+a_{0}}{S^{\beta }{\left(1-℘S^{-\alpha }\right)}^{\gamma }}}}\},\\ & =L^{-1}\{\sum_{k=0}^{\infty }\sum_{n=0}^{\infty }\sum_{m=0}^{n}\frac{{\left(\varphi -2k-2\right)}^{n}{\left(a_{0}\right)}^{n/2-m}\Gamma \left(n/2+1\right)}{n!m!\Gamma (n/2-m+1)}\frac{1}{S^{\left(\beta n/2-m\right)}{\left(1-℘S^{-\alpha }\right)}^{\gamma n/2}}\},\\ & =\sum_{k=0}^{\infty }\sum_{n=0}^{\infty }\sum_{m=0}^{n}\frac{{\left(\varphi -2k-2\right)}^{n}{\left(a_{0}\right)}^{n/2-m}\Gamma \left(n/2+1\right)}{n!m!\Gamma (n/2-m+1)}t^{\beta n/2-m-1}E_{\alpha ,\beta n/2-m}^{\gamma n/2}\left(℘t^{\alpha }\right), \end{array}$$ (23)

$$\begin{array}{cc} \Omega (t) & =L^{-1}\{\frac{Q_{0}e^{-b_{0}\varphi S}}{(\left(S+a_{0}\right)-b_{0}^{2}S^{\beta +2}{\left(1-℘S^{-\alpha }\right)}^{\gamma })\sqrt{\frac{S+a_{0}}{S^{\beta }{\left(1-℘S^{-\alpha }\right)}^{\gamma }}}}\},\\ & =L^{-1}\{e^{-b_{0}\varphi S}\sum_{k=0}^{\infty }\frac{Q_{0}b_{0}^{2k}S^{k(\beta +2)}{\left(1-℘S^{-\alpha }\right)}^{\gamma k}}{{\left(S+a_{0}\right)}^{k+1}}\},\\ & =Q_{0}\delta \left(t-b_{0}\varphi \right)*\sum_{k=0}^{\infty }{\left(b_{0}\right)}^{2k}\frac{e^{-a_{0}t}t^{k}}{\Gamma (k+1)}*t^{-k(\beta +2)-1}E_{\alpha ,-k(\beta +2)}^{\gamma k}\left(℘t^{\alpha }\right), \end{array}$$ (24)

$$\begin{array}{cc} D(t) & =L^{-1}\{\bar{D}\left(S\right)\},\\ & =L^{-1}\{\frac{Q_{m}}{S(S+a_{0})}\},\\ & =\frac{Q_{m}}{a_{0}}L^{-1}\{\frac{1}{S}-\frac{1}{S+a_{0}}\},\\ & =\frac{Q_{m}}{a_{0}}[1-e^{-a_{0}t}], \end{array}$$ (25)

The required solution of the model after employing the definition of inverse Laplace operator on the above Eq 18 is

$$\begin{array}{cc} T(\varphi ,t) & =[\sum_{m=0}^{\infty }b_{0}^{1+2m}Q_{0}t^{-(m+1/2)(\beta +2)-1}E_{\alpha ,-(m+1/2)(\beta +2)}^{-\gamma (m+1/2)}\left(℘t^{\alpha }\right)*e^{-a_{0}t}t^{m+1/2}]\\ & *[\sum_{k=0}^{\infty }\sum_{n=0}^{\infty }\sum_{m=0}^{n}\frac{{\left(-1\right)}^{n}{\left(1+2k+\varphi \right)}^{n}{\left(a_{0}\right)}^{n/2-m}\Gamma \left(n/2+1\right)}{n!m!\Gamma (n/2-m+1)}\\ & \times {\left(t-b_{0}\right)}^{\beta n/2-m-1}E_{\alpha ,\beta n/2-m}^{\gamma n/2}\left(℘{\left(t-b_{0}\right)}^{\alpha }\right)u\left(t-b_{0}\right)\\ & -[\sum_{k=0}^{\infty }\sum_{n=0}^{\infty }\sum_{m=0}^{n}\frac{{\left(-1\right)}^{n}{\left(2k+\varphi \right)}^{n}{\left(a_{0}\right)}^{n/2-m}\Gamma \left(n/2+1\right)}{n!m!\Gamma (n/2-m+1)}t^{\beta n/2-m-1}E_{\alpha ,\beta n/2-m}^{\gamma n/2}\left(℘t^{\alpha }\right)]]\\ & +[\sum_{m=0}^{\infty }b_{0}^{1+2m}Q_{0}t^{-(m+1/2)(\beta +2)-1}E_{\alpha ,-(m+1/2)(\beta +2)}^{-\gamma (m+1/2)}\left(℘t^{\alpha }\right)*e^{-a_{0}t}t^{m+1/2}]\\ & *[\sum_{k=0}^{\infty }\sum_{n=0}^{\infty }\sum_{m=0}^{n}\frac{{\left(\varphi -2k-1\right)}^{n}{\left(a_{0}\right)}^{n/2-m}\Gamma \left(n/2+1\right)}{n!m!\Gamma (n/2-m+1)}\\ & \times {\left(t-b_{0}\right)}^{\beta n/2-m-1}E_{\alpha ,\beta n/2-m}^{\gamma n/2}\left(℘{\left(t-b_{0}\right)}^{\alpha }\right)u\left(t-b_{0}\right)\\ & -\sum_{k=0}^{\infty }\sum_{n=0}^{\infty }\sum_{m=0}^{n}\frac{{\left(\varphi -2k-2\right)}^{n}{\left(a_{0}\right)}^{n/2-m}\Gamma \left(n/2+1\right)}{n!m!\Gamma (n/2-m+1)}t^{\beta n/2-m-1}E_{\alpha ,\beta n/2-m}^{\gamma n/2}\left(℘t^{\alpha }\right)]\\ & +[Q_{0}\delta \left(t-b_{0}\varphi \right)*\sum_{k=0}^{\infty }{\left(b_{0}\right)}^{2k}\frac{e^{-a_{0}t}t^{k}}{\Gamma (k+1)}*t^{-k(\beta +2)-1}E_{\alpha ,-k(\beta +2)}^{\gamma k}\left(℘t^{\alpha }\right)]+\frac{Q_{m}}{a_{0}}[1-e^{-a_{0}t}]. \end{array}$$ (26)

where $A\left(S\right)=\frac{S+a_{0}}{S^{\beta }{\left(1-℘S^{-\alpha }\right)}^{\gamma }}$, $\bar{B}\left(S\right)=\frac{Q_{0}}{\left(S+a_{0}\right)-b_{0}^{2}S^{\beta +2}{\left(1-℘S^{-\alpha }\right)}^{\gamma }}$, $\bar{E}\left(S\right)=\frac{b_{0}SB\left(S\right)}{\sqrt{A(S)}}$ and $\bar{D}\left(S\right)=\frac{Q_{m}}{S(S+a_{0})}$ Also, $L^{-1}\{\frac{1}{S^{\beta }{\left(1-℘S^{-\alpha }\right)}^{\gamma }}\}=L^{-1}\{\frac{S^{\alpha \gamma -\beta }}{{\left(S^{\alpha }-℘\right)}^{\gamma }}\}=t^{\beta -1}E_{\alpha ,\beta }^{\gamma }\left(℘t^{\alpha }\right)$.

## Results and discussion

**Fig 2:**
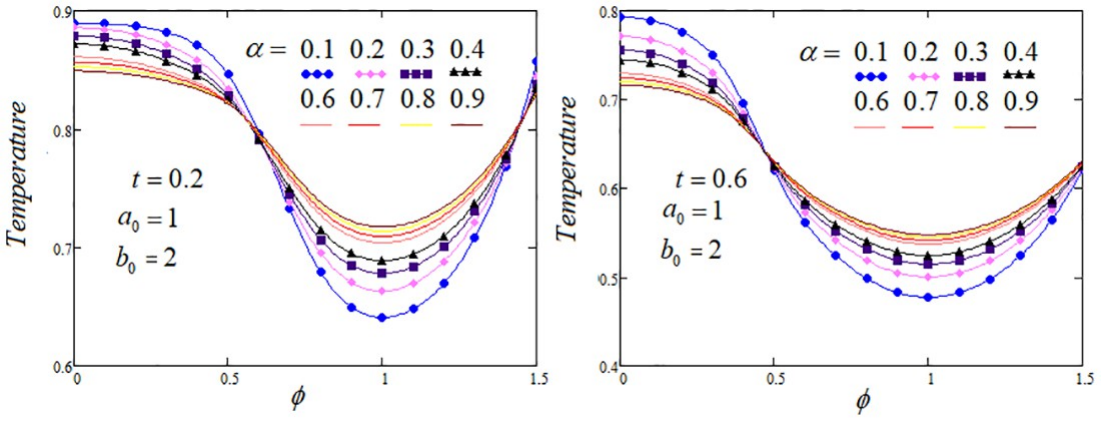
Temperature profile by varying the fractional parameter *α* when *Q*_*m*_ = 1.2 and *Q*_0_ = 1.

**Fig 3:**
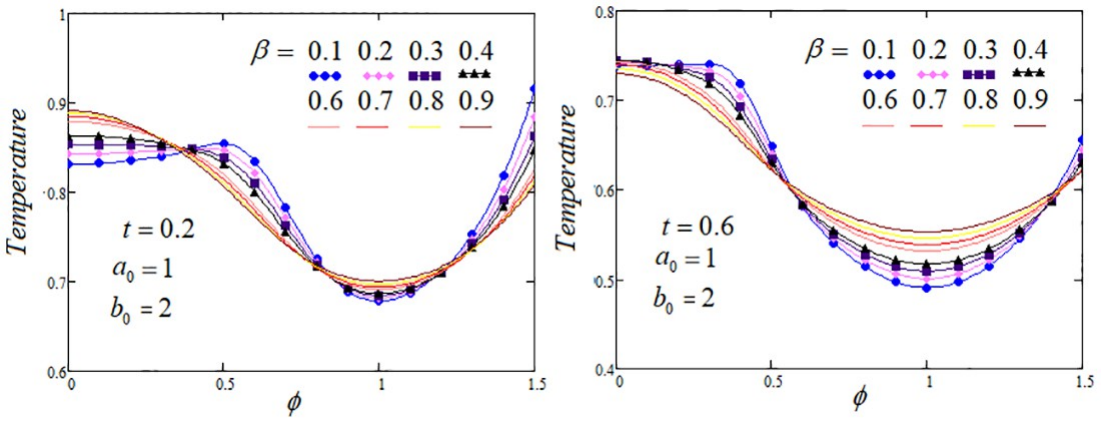
Temperature profile by varying the fractional parameter *β* when *Q*_*m*_ = 1.2 and *Q*_0_ = 1.

**Fig 4:**
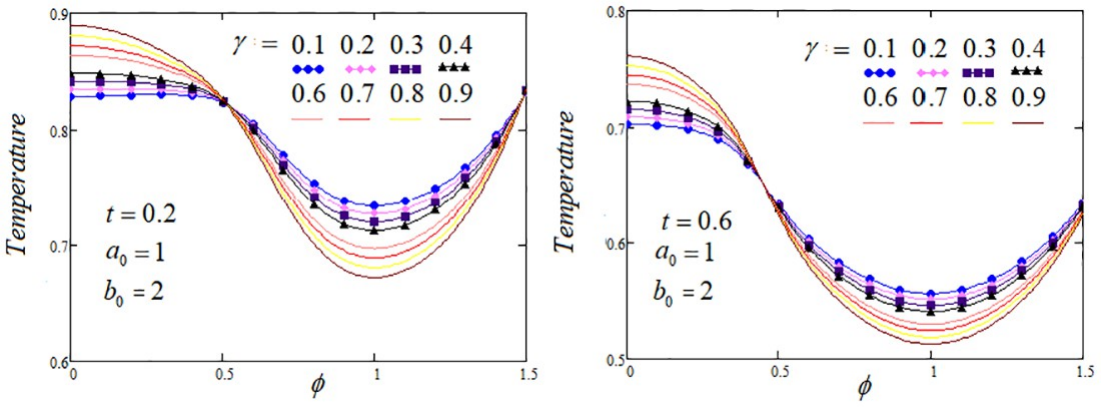
Temperature profile by varying the fractional parameter *γ* when *Q*_*m*_ = 1.2 and *Q*_0_ = 1.

**Fig 5:**
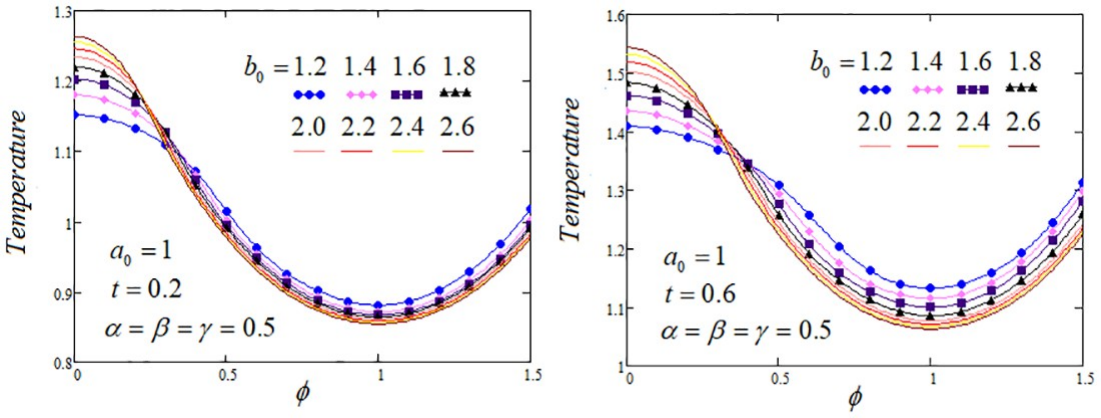
Temperature profile by varying the heat source parameter *b*_0_ when *Q*_*m*_ = 1.2 and *Q*_0_ = 1.

**Fig 6:**
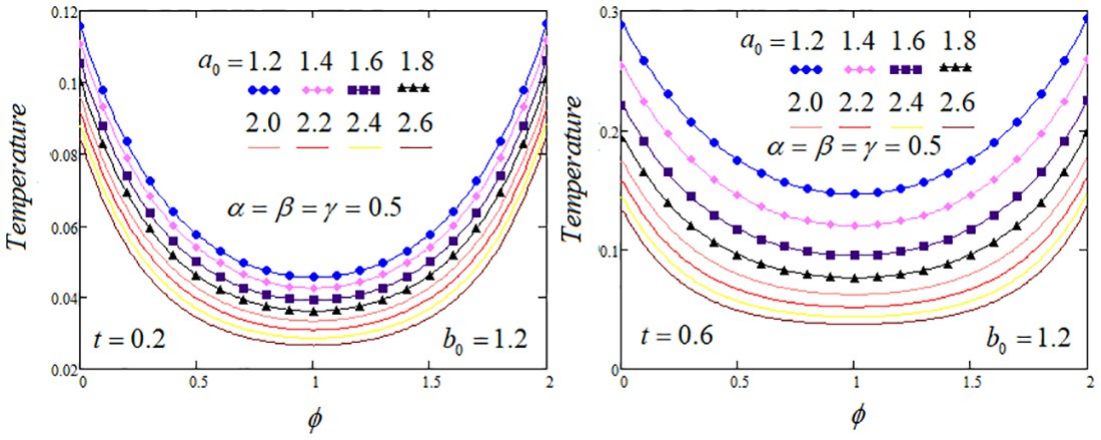
Temperature profile by varying the blood perfusion parameter *a*_0_ when *Q*_*m*_ = 1.2 and *Q*_0_ = 1.

**Fig 7:**
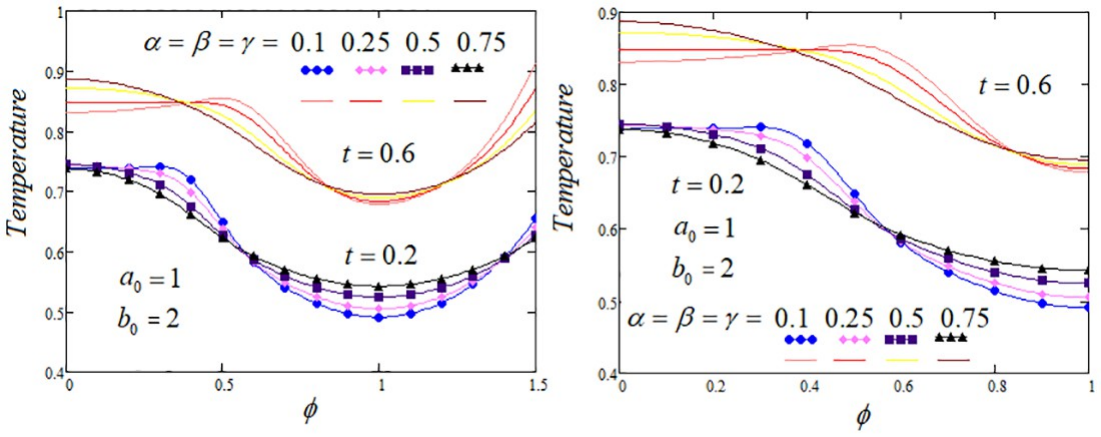
Temperature profile by taking the different values of time t corresponding to the same fractional parameters *α*,*β*,*γ* values, *Q*_*m*_ = 1.2 and *Q*_0_ = 1.

The heat transference analysis within biological tissues, we explore an extended version of the Pennes equation, that serves as a mathematical model. To understand the intricate thermal diffusion processes within biological tissues are the main goal of this research, by defining the thermal flux and develop a fractional differential equation involving the non-integer order derivative Prabhakar fractional operator. It’s crucial to acknowledge that the thermal flux isn’t solely dictated by the temperature gradient during thermal processes, but also takes into account its historical behaviour. Furthermore, in this research, it is investigated that heat transfer while considering the presence of a mobile heat source, that generates periodically thermal shocks at specific locations within the biological tissue field. To make our analysis more tractable, the dimensionless system of equations are employed to normalize the equation and the non-dimensional temperature phenomenon is solved by the integral LT. However, the expression for the non-dimensional temperature in the Laplace domain obtained in order to find the analytical solution via Prabhakar approach are more complex, due to the complexity of such expression, it is a big challenge to finding the inverse Laplace transform. Therefore, the obtained results are presented in a series form and also in the form of generalized special functions namely Mittag-Leffler function. The graphical illustration is used to present the behaviour of embedded system parameters in Figs 2–7 with the help of graphical software.

The heat transfer process analysis through the memory parameters which control on the temperature profile are carried out. To highlight the tissue temperature variation for distinct values of fractional parameters *α*, *β* and *γ* at two values of *t* are examined. It is analysed from the graphs that temperature profile elevated as enhancing the values of *β* and *γ*, but noticed that temperature profile descending, when the parameter *α* rises, The convenient choice of the fractional parameters *α*, *β*, *γ* and other system parameters that could lead to optimal cooling/heating of certain tissue areas. Further, in certain regions of the biological tissue, temperature profile exhibits oscillations or undergoes fluctuations, with periodic increases or decreases, these effects are elaborated in Figs 2–4.

Fig 5 exhibits the impact of the heat source parameter namely *b*_0_ at different time values of temperature profile. It is depicted from graph that rising the temperature curves as ascending the heat source parameter but the curves meet at a point and reverse behaviour noticed for two time levels. duration t of the plate.Temperature profiles are depicted at the center of the biological tissue. It is evident that varying the source parameter *b*_0_ by taking various values of involving parameters that leads to a shifting of the thermal shock’s position, resulting in oscillations corresponding to temperature values.

Fig 6 interpreted the the impact of *a*_0_ that represents blood perfusion, dictates the characteristics of the convective term (−*a*_0_*T*(*φ*, *t*)) in Eq (5). Consequently, this parameter exerts a substantial influence on the temperature profile. Also, from this figure of the graph, it is detected that curves against the fractional parameters, temperature profiles within the tissue as the values of parameter *a*_0_ rises. Notably, the values of the parameter *a*_0_ increases then the temperature profile decreases. This effect is attributed to the convective term acting as a cooling factor.

Fig 7 draw to illustrate variations in tissue temperature for two distinct time values and various different settings of the fractional parameters *α*, *β*, *γ*. It is examined that the peculiar shape of the heat source renders the dimensionless parameter *a*_0_ and *b*_0_, it is an important factor in shaping the temperature profile. Notably, within the biological tissue, a thermal shock is applied at the position *φ* at any time as vividly depicted in this graph, where the temperature distribution attained its maximum value at these positions. The judicious selection of the fractional parameters *α*, *β*, *γ* and the parameter *b*_0_ can potentially lead to optimize the cooling or heating of specific areas within the tissue.

## Conclusion

In this research article, an extended version of the time-fractional generalized Pennes bio-heat equation, that serves as a mathematical model, within biological tissues the heat transference analysis had been explored along with a constant metabolic heat generation source. To understand the intricate thermal diffusion processes within biological tissues are the main goal of this research, by defining the thermal flux namely the generalized Fourier’s law, and develop a fractional differential equation involving the non-integer order derivative Prabhakar fractional operator. It is investigated that heat transfer while considering the presence of a mobile heat source, that generates periodically thermal shocks at specific locations within the biological tissue field. To make our analysis more tractable, the dimensionless system of equations are employed to normalize the equation and the non-dimensional temperature phenomenon is solved by the integral Laplace transform (LT). However, the expression for the non-dimensional temperature in the Laplace domain obtained in order to find the analytical solution via Prabhakar approach are more complex, due to the complexity of such expression, it is a big challenge to finding the inverse Laplace transform. Therefore, the obtained results are presented in a series form and also in the form of special functions. The control of various nested parameters on heat profiles is studied to grasp the dynamics of the present model. By judiciously selecting parameters for the mobile source generating the thermal shock, it becomes feasible to selectively induce either heating or cooling in designated regions of the biological tissue. Consequently, the thermal source induces a linear thermal shock at specific moments and locations within the tissue.
